# Compulsive Scratching and the Psychoanalytic View of Repetition

**DOI:** 10.1192/j.eurpsy.2026.11057

**Published:** 2026-07-31

**Authors:** E. M. Jetter, D. Valle, J. Warner, G. Calero, B. R. Carr

**Affiliations:** 1 College of Medicine; 2 Department of Psychiatry, University of Florida, Gainesville, United States

## Abstract

**Introduction:**

The itch–scratch cycle in lichen simplex chronicus is familiar to every dermatologist: relief in the moment, harm in the long run, often recurring at the same sites. Freud described repetition compulsion as the return of what cannot be worked through (Freud S. Beyond the Pleasure Principle 1920). This framing adds an interpretive layer for how physical symptoms may carry unconscious meaning. Anzieu proposed the skin as a symbolic boundary of self, making dermatologic symptoms especially resonant for unconscious conflict (Anzieu D. The Skin-Ego 1989). Winnicott situated these dynamics in the developing self at the skin boundary, where integrity and vulnerability are felt (Winnicott DW. Ego Distortion in Terms of True and False Self 1960). These perspectives guide clinical engagement when scratching resists straightforward control.

**Objectives:**

To explore the itch–scratch cycle through psychoanalytic concepts of compulsion, boundary, and self-experience, and to consider implications for clinical care and medical education.

**Methods:**

A narrative review of psychoanalytic writings on repetition, boundary, and self-experience, alongside literature in dermatology and psychosomatic medicine, was conducted. Clinical descriptions of compulsive scratching were examined for their psychological as well as physiological meaning.

**Results:**

Scratching soothes and harms at the same time, a paradox that psychoanalysis captures in the concept of repetition compulsion. Patients often describe the act as irresistible and oddly relieving, yet also automatic and punishing, which helps explain why it is so difficult to stop. In psychoanalytic terms, the symptom not only reenacts unresolved conflict but also regulates fragile boundaries of self. Viewing the itch–scratch cycle in this way highlights its compulsive nature and opens clear opportunities for medical education. In teaching, it can be used as (1) a clinical example of how unconscious processes manifest in everyday symptoms, (2) a dermatology and psychiatry bridge showing how both perspectives are needed, and (3) a case discussion exercise where trainees practice recognizing psychological meaning in physical complaints and consider how this shapes communication with patients.

**Conclusions:**

The itch–scratch cycle shows how surface symptoms can embody unconscious dynamics of repetition and boundary. For clinicians, it clarifies why scratching often resists simple behavioral instruction and emphasizes the value of including psychodynamic perspectives alongside biomedical care. For educators, it offers a concrete micro-case to show how psychoanalytic thought adds interpretive depth without replacing biomedical care.

**Disclosure of Interest:**

None Declared

